# Effects of Combined Bacterial Infection and Radiation Injury on Biofluid Metabolite Profiles in the Murine Model

**DOI:** 10.1021/acsomega.5c06273

**Published:** 2025-10-15

**Authors:** Evan L. Pannkuk, Anika Kot, Lorreta Yun-Tien Lin, Igor Shuryak, Eric Wang, Albert J. Fornace, Heng-Hong Li

**Affiliations:** † Department of Oncology, Lombardi Comprehensive Cancer Center, 12231 Georgetown University Medical Center, Washington, D.C., District of Columbia 20057, United States; ‡ Department of Biochemistry and Molecular & Cellular Biology, Georgetown University Medical Center, Washington, D.C., District of Columbia 20057, United States; § Center for Metabolomic Studies, 8368 Georgetown University, Washington, D.C., District of Columbia 20057, United States; ∥ Center for Radiological Research, 5798 Columbia University Irving Medical Center, New York, New York 10027, United States

## Abstract

Rapid biodosimetry tools are needed to assess radiation exposure in scenarios complicated by secondary infections. This study evaluated how *Listeria monocytogenes* infection impacts metabolite-based biodosimetry in male C57BL/6 mice. The mice were infected and exposed to 0, 2, or 6 Gy X-rays at 4 days postinfection. Untargeted metabolomics was performed on serum and urine at 1 day postirradiation. We found that the effect of bacterial infection increased white blood cell counts and altered metabolomic signatures in a biofluid- and compound-specific manner. Infection alone altered select serum lipids and urinary TCA intermediates. Some urinary metabolites displayed additive effects in infected animals exposed to 6 Gy. The best model for combined biofluids (serum: lysophosphatidylcholines [14:0] and [22:5], glycerophosphatidylcholines [42:8] and [42:11] and citrate; urine: glutamic acid, creatine, propionylcarnitine, acetylspermidine, and hexanoylglycine) was determined with a multivariate random forest analysis model. A combined biofluid random forest model predicted the radiation dose and infection status with 90% accuracy (RMSE = 1.31 Gy). These findings support the development of robust, multiplexed biodosimetry panels capable of accounting for real-world confounders like infection. Such models can improve the precision of triage decisions following radiological emergencies (raw data available at Metabolomics Workbench Study IDs ST004101 and ST004100).

## Graphical abstract

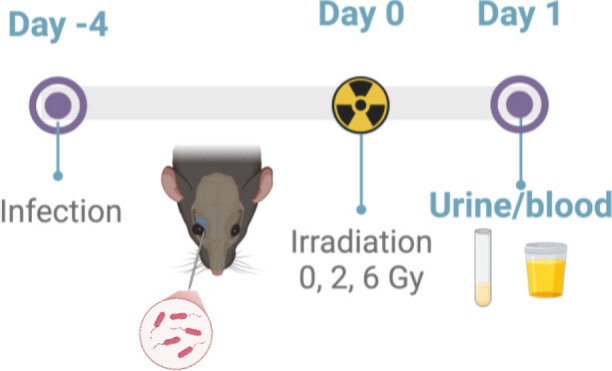

## Introduction

It is critical to have emergency response strategies in place to properly guide medical triage in potential radiological emergencies involving exposure of ionizing radiation (IR) to the general public. Typically, these types of emergencies will fall into two categories: intentional (e.g., terrorist threats) or accidental (e.g., nuclear power plant disasters). In either situation, high-throughput biodosimetry assays are needed to estimate the degree of acute radiation syndrome so that proper medical triage and countermeasures can be administered to a large group of people.^1^ In terms of developing novel high-throughput biodosimetry assays, we have focused on the use of noninvasive biofluids (e.g., urine, saliva, and blood) for the rapid analysis of small-molecule biomarkers using mass spectrometry (MS) platforms. Multiplex metabolite panels can then be combined with other biomarker types (gene/protein expression or complete blood counts (CBC)) to increase the accuracy of dose reconstruction.^2^ Multiple biomarkers will likely be required due to general heterogeneity in both the genetic makeup of the individuals exposed, aspects of a complex exposure (neutron vs gamma, partial body, etc.), and pre-exposure samples will not be available for comparison. In addition, changes in metabolite levels from combined injuries, infections, and burns may coincide with IR injury or exacerbate their change.^3,4^ Critical care of these injuries (reviewed here^5^), such as antibiotics, antihistamines, myeloid cytokines, and general supportive care, will also alter the endogenous metabolite levels in biofluids.

Acute radiation syndrome can result in impaired hematopoietic function and loss of intestinal lining, increasing susceptibility to infections due to neutropenia, and gut barrier dysfunction.^6^ While infections can be of bacterial, viral, or fungal origin, bacterial infections of the blood (bacteremia and septicemia) and lungs (pneumonia) will be of the highest concern in a nuclear emergency. In early studies using the murine model, it was shown that irradiated animals have a reduction in the normal gut microflora and weakened gut barrier integrity, followed by bacterial translocation from the gut to the bloodstream, leading to septic shock, and lethality.^7–10^ Microbiome analysis in mice following 5 or 12 Gy IR exposure highlighted that there was a differential effect during the following 30 days, where *Lactobacillaceae* and *Staphylococcaceae* bacteria increased but reduced abundance was observed for *Ruminococcaceae*, *Clostridiaceae*, and *Lachnospiraceae*.^11^ Higher maintenance of *Enterococcaceae* and *Lachnospiraceae* was postulated to play a role in survival for mice exposed to 9.2 Gy.^12^ These microbiota shifts, combined with secondary injury and supportive care, highlight the complex nature of nuclear events and underscore the need for specialized animal models in developing medical countermeasures and biodosimetry assays.^13^

One well-established animal model of bacterial infection is the murine model infected with *Listeria monocytogenes*, which has been utilized as a model pathogen since the 1960s^14^ and elicits one of the most well-understood mammalian immune responses established in the literature.^15^ *L. monocytogenes* is a Gram-positive food-borne pathogen that invades cells through the secretion of the toxin listeriolysin O (LLO), which forms a pore allowing entry into the cytosol where it replicates, then becomes motile, and disseminates across other organs and cell types, inducing the MHC class I pathway to initiate a strong CD8^+^ T cell response. Infection with the recombinant strains of *L. monocytogenes*, such as strains that overexpress OVA (*Lm-OVA*), display acute proliferation and antigen presentation in vivo compared to nonrecombinant *L. monocytogenes*.^16^ Early innate inflammatory responses include inflammasome activation and cytokine release as *L. monocytogenes* is cleared from the circulation. At 4 dpi, high bacteria burden is typically observed in spleen and liver, along with features of a robust innate and adaptive immune response in intravenously infected mice, including the activation of innate immune cells, expansion of CD8^+^ T cells, and production of inflammatory cytokines.^15,17^

It is well established that many IR exposure victims will have associated injuries and develop microbial infections following a radiological emergency. Yet studies on the combined effects of IR exposure, infectious diseases, and medical countermeasures are less explored, especially in the context of biodosimetry sensitivity. In this study, we examine changes in biofluid metabolite concentrations using the C57BL/6 murine model due to bacterial infection combined with radiation injury. Specifically, we infected mice with a retro-orbital injection of *Lm-OVA* and analyzed urine and serum in male mice at 1 d (5 dpi) after a 2 or 6 Gy X-ray dose (infect + IR-exposed mice) using untargeted metabolomics. Sham-irradiated mice infected with *Lm-OVA* (infect + sham), sham-irradiated uninfected mice (noninfect + sham), and irradiated uninfected mice (noninfect + IR) were included as controls. We found that the effects of a bacterial blood infection on radiation metabolites were biofluid- and compound-specific, with an attenuated effect observed in serum, as infection alone decreased several lipid levels without IR exposure. Conversely, in urine, the combination of infection and 6 Gy IR exposure appeared to have a cumulative effect with increased fold changes in several metabolites, excluding TCA cycle intermediates, Hex-V-I, and azelaic acid. As in previous studies, we show that multiplex panels of radiation response metabolites in biofluids can be combined to give excellent sensitivity and specificity to identify irradiated individuals and provide invaluable biomarkers for novel biodosimetry assays.

## Materials and Methods

### Animal Models and Radiation Exposure

All animal experiments were approved by the Georgetown University Institutional Animal Care and Use Committee (IACUC, protocol #2023-0012) and were conducted under all relevant federal and state guidelines. Male C57BL/6 mice (10 weeks old) were purchased from Charles River Laboratories (Frederick, MD) and provided food (PicoLab Rodent Diet 20 #5053), and deionized water was provided ad libitum. The mice were infected with a retro-orbital injection of *Lm-OVA*, which is a widely used and reproducible infection method as the bacteria can bypass the gut stage of infection. *Lm-OVA* stocks frozen at −80 °C were grown overnight at 37 °C while being shaken in brain heart infusion (BHI) broth supplemented with 5 μg/mL erythromycin. Then, the overnight cultures were subcultured by diluting into fresh BHI broth supplemented with 5 μg/mL erythromycin and grown for 4 h at 37 °C while shaking. Bacterial cfu was then quantified by measuring optical density at 600 nm. For primary infections, bacterial culture was then diluted to 1 × 10^5^ cfu/100 μL in sterile 1× PBS, and 100 μL was injected per mouse. Both noninfected and infected mice were randomly assigned to a zero-dose sham (0 Gy) or irradiated mice (2 or 6 Gy) (Figure 1). We exposed mice to total body irradiation (TBI) in an acrylic 12-slot mouse pie cage (MPC-1, Braintree Scientific, Braintree, MA) using a specimen turntable (XD1905-0000, Precision X-ray Inc., Branford, CT). The mice were exposed to 0, 2, or 6 Gy X-ray IR (1.67 Gy/min; X-Rad 320, Precision X-ray Inc.; filter, 0.75 mm tin/0.25 mm copper/1.5 mm aluminum). At 1 d postirradiation, spot urine samples were collected; blood was collected via cardiac puncture; and serum for metabolomics was separated using BD Microtainer Tubes (REF 365967), with ∼100 μL of whole blood added to each tube, kept at room temperature for 30 min, and then centrifuged (1300*g*, 4 °C) for 10 min. Biofluids were flash-frozen and stored at −80 °C until analysis. A separate aliquot of blood was collected in a dipotassium EDTA tube (BD Cat #365974) for collecting CBC (including levels of white blood cells [WBC], lymphocyte [LYM], monocyte [MON], and neutrophil [NEU]) by VRL Diagnostics (Gaithersburg, MD, http://www.vrlsat.com/).

**Figure 1:**
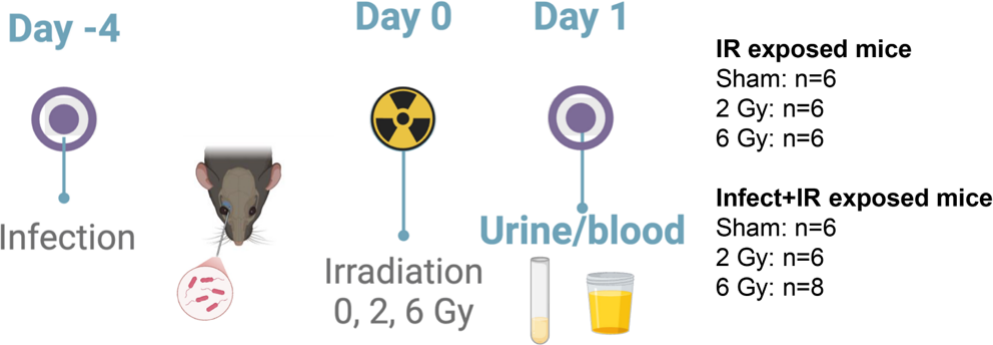
Sample sizes and experimental setup.

### Chemicals

The solvents for sample preparation and LC mobile phases were Optima brand reagents from Fisher Scientific (Hanover Park, IL, USA). Internal standards for both urine and serum were purchased from Sigma-Aldrich (St. Louis, MO, USA) (chlorpropamide and a deuterated amino acid standard mixture [SMB00917]). Chemical standards for validations including creatine, propionyl-l-carnitine, acetylcarnitine, azelaic acid, nicotinic acid, l-glutamic acid, citric acid, ketoglutaric acid, malic acid, and taurine were obtained from Sigma-Aldrich (St. Louis, MO, USA). Lysophosphatidylcholine (LysoPC) (14:0) was obtained from Avanti Polar Lipids Inc. (Alabaster, AL, USA). Hexosamine-valine-isoleucine-OH (Hex-V-I) and its deuterated isomer (Hex-V-I-d10) was synthesized by Expert Synthesis Solutions (London, ON, Canada).^18^ N1- and N8-Acetylspermidine, hexanoyl glycine, and PC (16:0/22:6) were obtained from Cayman Chemical Co. (Ann Arbor, MI). We used both NIST plasma Standard Reference Material (SRM) 1950 (plasma) and 3667 (urine) that were produced by NIST as quality control (QC) samples (Gaithersburg, MD, USA).

### Untargeted Metabolite Profiling in Biofluids

Biofluids were prepared as previously described, where we use a standard “dilute-and-shoot” protocol.^19^ A 20 μL aliquot of urine was mixed with cold 50% acetonitrile (ACN) (80 μL) containing an internal standard (5 μM chlorpropamide [M + H]^+^ = 277.0414, [M – H]^−^ = 275.0257). We prepared the samples on ice with vortexing and 10 min incubation periods following each step. The samples were then centrifuged at maximum speed for 10 min (10,000*g*, 4 °C), with aliquots then being transferred to a liquid chromatography (LC) vial for analysis. For serum, a 5 μL aliquot was mixed with 195 μL of cold 66% acetonitrile containing internal standards (5 μM chlorpropamide [M + H]^+^ = 277.0414, [M – H]^−^ = 275.0257; deuterated amino acid mix). The deuterated amino acid mix contains 24 different labeled metabolites, ranging in concentration from 222 μM for glutamine-d5 to 7 μM for 1-methylhistidine-d3. The cold 66% acetonitrile used for metabolite extraction contained the amino acid mixture adjusted to a concentration of 10 μM for glutamine-d5. Samples were then prepared as described above for urine. 1 μL aliquots of each biofluid sample were combined as a QC sample. Additional QC samples included the creatinine in frozen human urine and metabolites in frozen human plasma NIST Standard Reference Materials. The QC samples were injected every 10 samples along with blanks.

Samples were injected (2 μL) into a Waters Acquity Ultra-Performance Liquid Chromatography (UPLC) BEH C18 1.7 μm, 2.1 mm × 50 mm column coupled to a Xevo G2S quadrupole time-of-flight (QTOF) MS (Waters, Milford, MA, USA) system, as previously described,^20^ with data collected in both positive and negative electrospray ionization (ESI) modes for urine and serum using data-independent acquisition, with leucine enkephalin used for Lock-Spray ([M + H]^+^ = 556.2771, [M – H]^−^ = 554.2615). Our ESI operating conditions for both biofluids were set at a capillary voltage of 2.0 kV, cone voltage of 30 V, source temperature of 120 °C, desolvation temperature of 500 °C, desolvation gas flow of 1000 L/h. Mobile phases for urine were: solvent A (water/0.1% formic acid [FA]), solvent B (acetonitrile/0.1% FA). Gradient: (solvent A and B) 4.0 min 5% B, 4.0 min 20% B, 5.1 min 95% B, and 1.9 min 5% B at a flow rate of 0.5 mL/min, column temp 40 °C. Mobile phases for serum were: solvent A (water/0.1% FA), solvent B (acetonitrile/0.1% FA), solvent C (isopropanol/0.1% FA). Gradient: (solvent A and B) 4.0 min 2% B, 4.0 min 60% B, and 1.5 min 98% B. The wash phase was 2 min 11.8% B and 88.2% C, followed by re-equilibration at 98% A and 2% B with a flow rate of 0.5 mL/min, column temperature of 60 °C.

### Data Processing, Statistical Analysis, and Marker Validation

Raw data files were manually inspected in MassLynx v.4.1 (Waters Corporation, Milford, MA, USA) and then imported into Progenesis QI (Nonlinear Dynamics, Newcastle, UK) for preprocessing, including peak alignment and picking. Data were normalized to the “normalize to all compounds function” for both biofluids. The putative identifications for spectral features were determined (±10 ppm error) of the monoisotopic mass using the Human Metabolome Database (HMDB)^21^ and the METLIN MS/MS empirical library^22^ databases. After the spectral features were matched to an accurate *m*/*z* (<10 ppm for the precursor ion and <30 ppm for product ions) for initial identification, the retention time and the tandem MS (5–50 V ramping collision energy) fragmentation pattern were matched to a pure standard for assignment to a metabolomics standards initiative (MSI) level 1.^23^ The retention times of serum amino acids were matched to their respective spiked deuterated standard. The tandem MS spectra from both the sample and standard were then inspected using a reference spectrum in the NIST/EPA/NIH Mass Spectral Library 20 v.2.4 when available. Lipids are typically identified to a MSI level 2, where a representative authentic standard from each group (PC (16:0/22:6) or LysoPC (14:0)) is run, and then they are identified based on their fragmentation and relative retention time from the acyl chain length and degree of unsaturation.

The data matrices for urine and serum was assessed individually by ranking the spectral features by false discovery rate corrected p values in MetaboAnalyst 6.0, validated compounds of interest were graphed and checked for outliers (ROUT Q = 1%), equal variances (Bartlett’s test), and normal distributions (Shapiro–Wilk test) in GraphPad Prism 9.2.0 (GraphPad Software, La Jolla, CA, USA). Compounds with equal variances and normal distributions were compared with standard one-way ANOVAs. Compounds that did not pass the equal variance test but had a normal distribution were compared with Welch’s ANOVA, and two compounds with non-normal distributions for more than 2 treatment groups (azelaic acid and malic acid) were compared with a Brown–Forsythe test. Heatmaps were generated in MetaboAnalyst 6.0 using the ANOVA function, and principal component analysis (PCA) plots were plotted after log transformation and Pareto scaling of the data matrices.^24^ The area under the curve (AUC) values were derived from receiver operating characteristic (ROC) curves generated using the Random Forests classification method in MetaboAnalyst 6.0. Pathway analysis was performed using MetaboAnalyst 6.0 with the *Mus musculus* SMPDB pathway library. CBC data were compared with a two-way ANOVA with Sidák’s multiple comparison test in GraphPad Prism 9.2.0.

To determine the top 10 metabolites from urine and serum that jointly predict the radiation dose and infection status in mice, we conducted a multivariate random forest analysis using the rfsrc­() function from the RandomForestSRC R package (v3.1.1) on the validated metabolites. After merging and preprocessing the validated urine and serum metabolomics data sets, including a *z*-score normalization (standardization to zero mean and unit variance based on the training set) and removal of non-numeric predictors, we trained a random forest model with optimized parameters to assess the joint predictive performance. All modeling and visualization steps were performed in R (version 4.4.1).

## Results and Discussion

In the event of a nuclear emergency, high-throughput assays will be needed to screen individuals, determine the extent of radiation injury, and guide medical triage and countermeasure administration. When using endogenous compounds (e.g., metabolites, proteins, and genes) to serve as a proxy for IR injury, several concurrent casualty events will contribute to these measurements that include wounds, trauma, burns, and infections, among others. The paucity of information from human subjects involving bacterial infections and IR exposure requires the development of animal models to estimate the precision of biodosimetry models. The murine model of *L. monocytogenes* infection is one of the most comprehensive models of infection and allows for a well-controlled system for initial testing of the combined effects from infection and IR exposure. We irradiated mice at 4 dpi, when *Lm-OVA* will still have a high bacterial burden in radiosensitive hematopoietic tissues, including the bone marrow, spleen, and liver.^25,26^ We found that the effects on IR biomarkers during *Lm-OVA* infection are highly dependent on the biofluid of interest, with infect + IR mice showing enhanced fold changes in the urinary metabolite response but an attenuated response in serum lipids.

### Radiation Metabolites in Urine

For urine, our initial data matrix was combined from 5806 spectral features in the ESI+ mode and 5944 spectral features in the ESI– mode. We plotted the individual samples using a PCA plot to determine the influence of *Lm-OVA* infection on spatial distributions following TBI doses of 2 and 6 Gy (Figure 2 and Table 1). We found that irrespective of infection, a clear separation of the 6 Gy groups was observed from 0 and 2 Gy groups. Although the interpretation of these multidimensional models may be difficult to translate to the clinic, they are useful in biomarker development for quick visualization of complex data sets. Here, we see that mice exposed to 6 Gy, representing the individuals needing immediate medical care, can be readily identified irrespective of prior infection.

**Figure 2:**
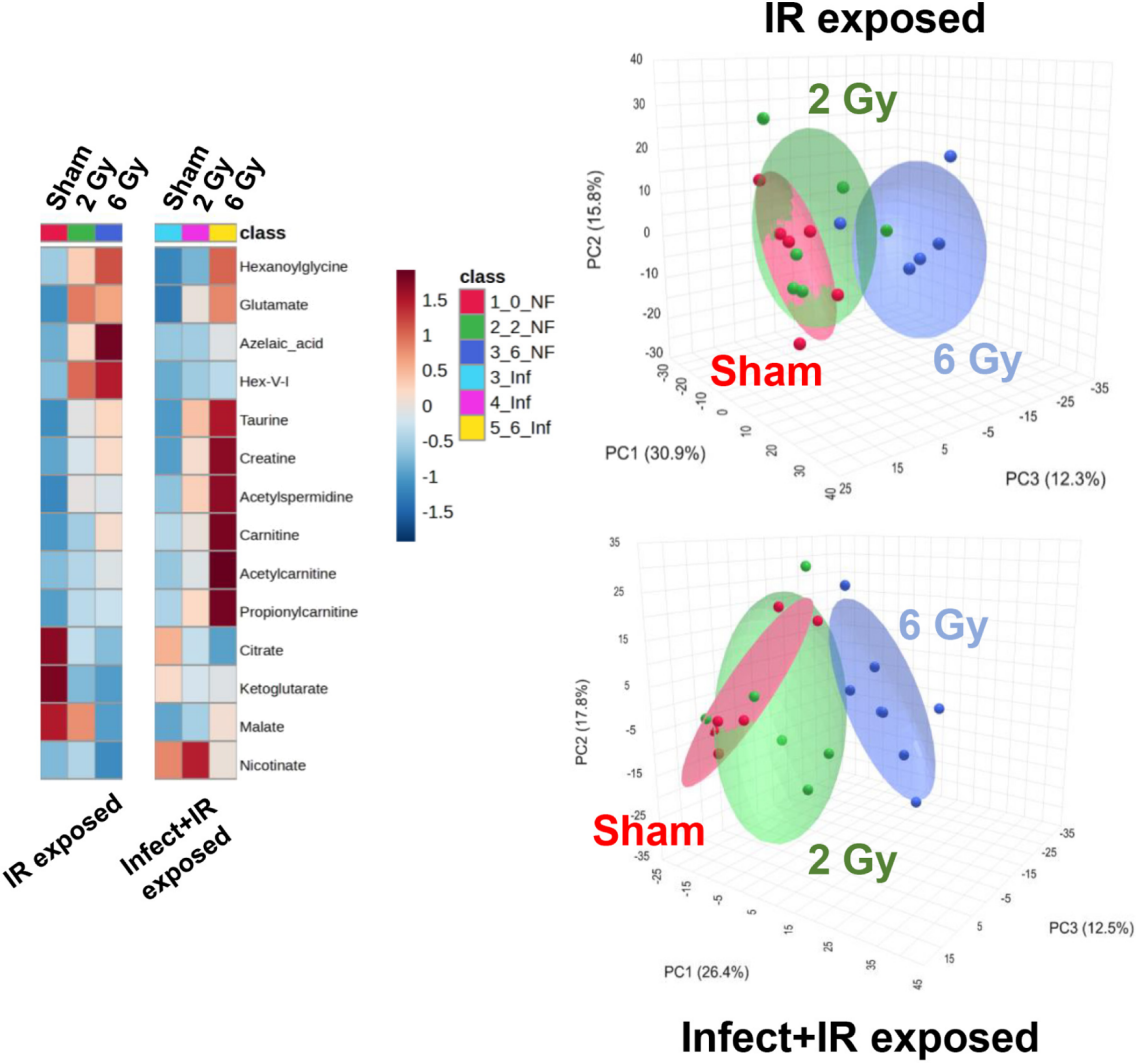
(Left) Heatmap showing fold changes of validated urine metabolites following IR exposure alone or in combination with *L. monocytogenes* infection. (Right) PCA of the combined ESI+ and ESI– data sets from untargeted metabolomic profiling showing that individuals exposed to 6 Gy IR separated from sham and 2 Gy irradiated individuals irrespective of the infection status.

**Table 1:** Validated Urinary Metabolites

								MS/MS fragments		
metabolite	adduct	RT	experimental (*m*/*z*)	calculated (*m*/*z*)	mass error (ppm)	HMDB	formula	fragment 1	fragment 2	fragment 3
Hex-V-I	H+	1.31	393.2228	393.2234	1.5	162421477^a^	C_17_H_32_N_2_O_8_	309.1802	216.124	150.0926
creatine	H+	0.30	132.0779	132.0773	4.5	0000064	C_4_H_9_N_3_O_2_	114.0657	90.0551	87.0564
carnitine	H+	0.31	162.1139	162.1130	5.5	0000062	C_7_H_16_NO_3_	103.0401	85.0294	60.0787
acetylcarnitine	H+	0.31	204.1236	204.1236	0.0	0000201	C_9_H_17_NO_4_	145.0506	85.0292	60.0818
propionylcarnitine	H+	0.41	218.1384	218.1392	3.7	0000824	C_10_H_19_NO_4_	159.0645	85.0291	60.0811
acetylspermidine	H+	0.27	188.1761	188.1763	1.1	0001276	C_9_H_21_N_3_O	171.1493	117.1033	100.0764
nicotinic acid	H+	0.64	124.0407	124.0399	6.4	0001488	C_6_H_5_NO_2_	106.027	96.0445	80.0456
glutamic acid	H–	0.30	146.0455	146.0458	2.1	0000148	C_5_H_9_NO_4_	102.0561	85.0296	59.0124
hexanoylglycine	H–	3.06	172.0973	172.0974	0.6	0000701	C_8_H_15_NO_3_	146.9651	128.1080	74.0237
azelaic acid	H–	4.40	187.0976	187.0970	3.2	0000784	C_9_H_16_O_4_	169.0857	125.0968	97.0686
citric acid	H–	0.31	191.0193	191.0192	0.5	0000094	C_6_H_8_O_7_	173.0089	111.0089	87.0084
malic acid	H–	0.30	133.0142	133.0137	3.8	0000156	C_4_H_6_O_5_	115.0035	89.024	71.0131
α-ketoglutaric acid	H–	0.30	145.0128	145.0137	6.2	0000208	C_5_H_6_O_5_	101.0239	73.0261	57.0331
taurine	H–	0.28	124.0072	124.0068	3.2	0000251	C_2_H_7_NO_3_S	106.9806	94.9803	79.9569

a Pubchem CID.

Statistically significant differences due to infection alone (i.e., between noninfect + sham and infect + sham) were highest for nicotinic acid (*p* = 0.014) and citric acid (*p* = 0.014) but were also observed for acetylspermidine (*p* = 0.021), propionylcarnitine (*p* = 0.046), and malic acid (*p* = 0.046) (Table 2). The significant increase in nicotinic acid due to infection is interesting, as this is not significantly increased using the LPS murine model of sepsis.^3^ However, we found a surprisingly minor metabolite perturbation due to *Lm-OVA* infection at 5 dpi. The highest fold changes were due to IR exposure (i.e., between noninfect + sham vs noninfect + IR or infect + sham vs infect + IR comparisons), with perturbations observed in pathways commonly associated with radiation response, including fatty acid metabolism (*p* = 0.002) and the TCA cycle (*p* = 0.048) (Figure S1). There were no missing values or outliers detected within the validated urinary compounds listed in Table 1. Overall, urinary metabolite profiles exhibited two primary yet distinct trends when examining the effects of bacterial infection on IR response: (1) *Lm-OVA* infection had a significant effect on the metabolite concentration that masked the IR effects (e.g., citric acid or malic acid) or (2) *Lm-OVA* infection did not significantly affect the metabolite concentration and in fact had a synergistic effect on its response following IR exposure (e.g., acetylcarnitine, taurine, and creatine) (Figure 2 and S2).

**Table 2:** Fold Changes and ANOVA *p* Values for Validated Urine Metabolites for IR-Exposed Noninfected or Infected Mice^a^

	irradiated						nonirradiated	
	*p* value		fold change				*p* value	fold change
	noninfected	infected	noninfected		infected		noninfected vs infected	
metabolite urine			2 Gy	6 Gy	2 Gy	6 Gy	sham	
Hex-V-I	<0.001	NS	3.0	3.6	1.4	1.7	0.687	0.8
creatine	0.034	0.002	1.4	1.6	1.6	2.5	0.774	1.0
carnitine	0.001	0.037	1.3	1.8	1.2	2.1	0.065	1.4
acetylcarnitine	NS	0.036	1.3	1.5	1.4	2.9	0.585	1.1
propionylcarnitine	0.002	0.019	1.5	1.6	1.4	2.3	0.046	1.4
acetylspermidine	0.008	<0.001	2.0	1.9	1.6	2.4	0.021	1.4
nicotinic acid	NS	0.018	1.1	0.9	1.1	0.8	0.014	1.5
glutamic acid	0.041	0.016	1.7	1.6	1.5	1.8	0.774	0.9
hexanoylglycine	0.019	<0.001	1.3	1.6	1.2	2.0	0.253	0.8
azelaic acid	0.020	NS	1.4	1.9	1.0	1.2	0.774	1.1
citric acid	<0.001	0.006	0.6	0.5	0.8	0.6	0.014	0.7
malic acid	NS	NS	0.8	0.4	1.2	1.6	0.046	0.4
α-ketoglutaric acid	0.018	NS	0.4	0.4	0.9	0.9	0.186	0.6
taurine	0.053	<0.001	1.4	1.6	1.6	2.0	0.774	1.0

a Statistically significant differences between the sham and infect + sham groups were also compared.

As expected, common IR-related urinary metabolites were identified in the noninfect + IR group. These included Hex-V-I and TCA cycle intermediates (ketoglutaric acid, citric acid, and malic acid), azelaic acid, and glutamic acid (Figures 2, S2, Tables 1 and 2). Hex-V-I is a novel metabolite previously described by our group^18^ that we later identified as having an IR-specific increase in urine that was independent of partial body exposures^20^ or dose rate.^27,28^ The source of Hex-V-I remains unknown, as it does not appear to be microbially produced by the primary host microbiota and increases in mice given broad-spectrum antibiotics,^29^ and is not detected in tissues post-IR (adipose, brain, colon, ileum, kidney, liver, lungs, muscle, serum, spleen, testes, bladder, and stomach) (unpublished data). Consistent with previous studies, we found higher fold changes post-IR for urinary Hex-V-I compared to other IR biomarkers (noninfect + IR 2 Gy FC = 3.0, 6 Gy = 3.6), although its concentration is statistically unchanged in *Lm-OVA-*infected mice (infect + IR 2 Gy FC = 1.4, 6 Gy = 1.7) (Table 2). Azelaic acid is a dicarboxylic acid (DCA) polyamine that shows time-dependent increases in hair samples^30^ after IR exposures and has also been identified in urine^31,32^ and serum samples.^33^ Azelaic acid has been widely studied in dermatology for its role in skin health,^34^ DCAs in biofluids have been reported following various complex IR exposures (internal emitters^35^ and partial body very high dose rate^20^) but can also be diet-related^31^ or artifacts from plasticizers.^36^ As these sources should be carefully considered,^37^ the dose- and time-dependent increases observed in urine (current study) and hair^30^ suggest that sources can be from systemic perturbation rather than suppressed appetite following IR exposure or sample storage.

Considering the TCA cycle intermediates (α-ketoglutaric acid, citric acid, and malic acid), there was a striking decrease in infect + sham mice (statistically significant for citric acid [*p* = 0.014] and malic acid [*p* = 0.046]) (Figures 2, S2 and Table 2). Decreased urinary TCA cycle intermediates, commonly citric acid,^38^ are typically detected following IR exposure.^19,39,40^ However, the decreased levels due to *Lm-OVA* infection obscured the IR effects, making them less effective for identifying exposed individuals. As previously discussed for DCAs, suppressed appetite following IR exposure could also play a role in differing TCA cycle intermediate concentration.^41^ The use of citric acid as a ubiquitous food and beverage additive needs to be considered in measurements from nonfasting humans.^42^ However, we found that glutamic acid was a more robust marker and was not affected by infection. Glutamic acid contributes to the TCA cycle via conversion to α-ketoglutaric acid. This urinary amino acid was retained as the top marker for ROC curve construction for urine alone and for both biofluids combined.

Interestingly, several common radiation urinary metabolites (creatine, acetylcarnitine, carnitine, propionylcarnitine, acetylspermidine, taurine, and nicotinic acid) had a higher fold change in mice with combined IR exposure and bacterial infection when compared to IR injury alone. Although the source of these metabolites in urine may not be immediately clear, the >2-fold increase in urine after IR exposure argues against these being due to suppressed appetite. As *Lm-OVA* invades liver cells and hematopoietic tissues, perturbation to fatty acid oxidation in addition to the TCA cycle occurs.^43^ These metabolic disruptions to fatty acid oxidation by *Lm-OVA* infection may act synergistically with the observed effects of IR exposure to the carnitine shuttle, thus explaining the drastic increases in free carnitine and urinary acylcarnitine.^44–46^ Regardless of potential synergistic effects, these metabolites distinguish individuals receiving a high IR dose (here, 6 Gy in the murine model) from those individuals receiving no exposure or not requiring immediate medical care (here, 0 or 2 Gy in the murine model). Further experiments could elucidate whether this effect could misclassify individuals as receiving very high total doses that are fatal irrespective of medical treatment.

The area under the receiver operating characteristic (AUROC) values indicate that our marker panel had high sensitivity and specificity for classifying irradiated individuals, similar to the results reported in previous studies.^47^ That is, the IR metabolite signature is predictive and repeatable enough to have high predictive results irrespective of treatment, such as dose rate, partial body exposure, or here, bacterial infection. We achieved excellent sensitivity and specificity for noninfect + sham vs that for noninfect + 6 Gy (AUROC = 1.0, CI = 1–1), infect + sham vs infect + 6 Gy (AUROC = 1.0, CI = 1–1), and infect + sham/2 Gy vs noninfect + 6 Gy (AUROC = 0.98, CI = 0.86–1) to very good sensitivity and specificity for noninfect + sham/2 Gy vs infect + 6 Gy (AUROC = 0.88, CI = 0.70–1) using a panel of 11 metabolites (carnitine, acetylcarnitine, propionylcarnitine, creatine, Hex-V-I, acetylspermidine, citric acid, hexanoylglycine, azelaic acid, glutamic acid, and taurine) previously identified in radiation metabolomics studies (Figure 3). Although the most consistently reported postirradiation metabolites have included carnitine, TML, creatine, and Hex-V-I, increased acetylcarnitine has also been validated in NHP urine.^19^

**Figure 3:**
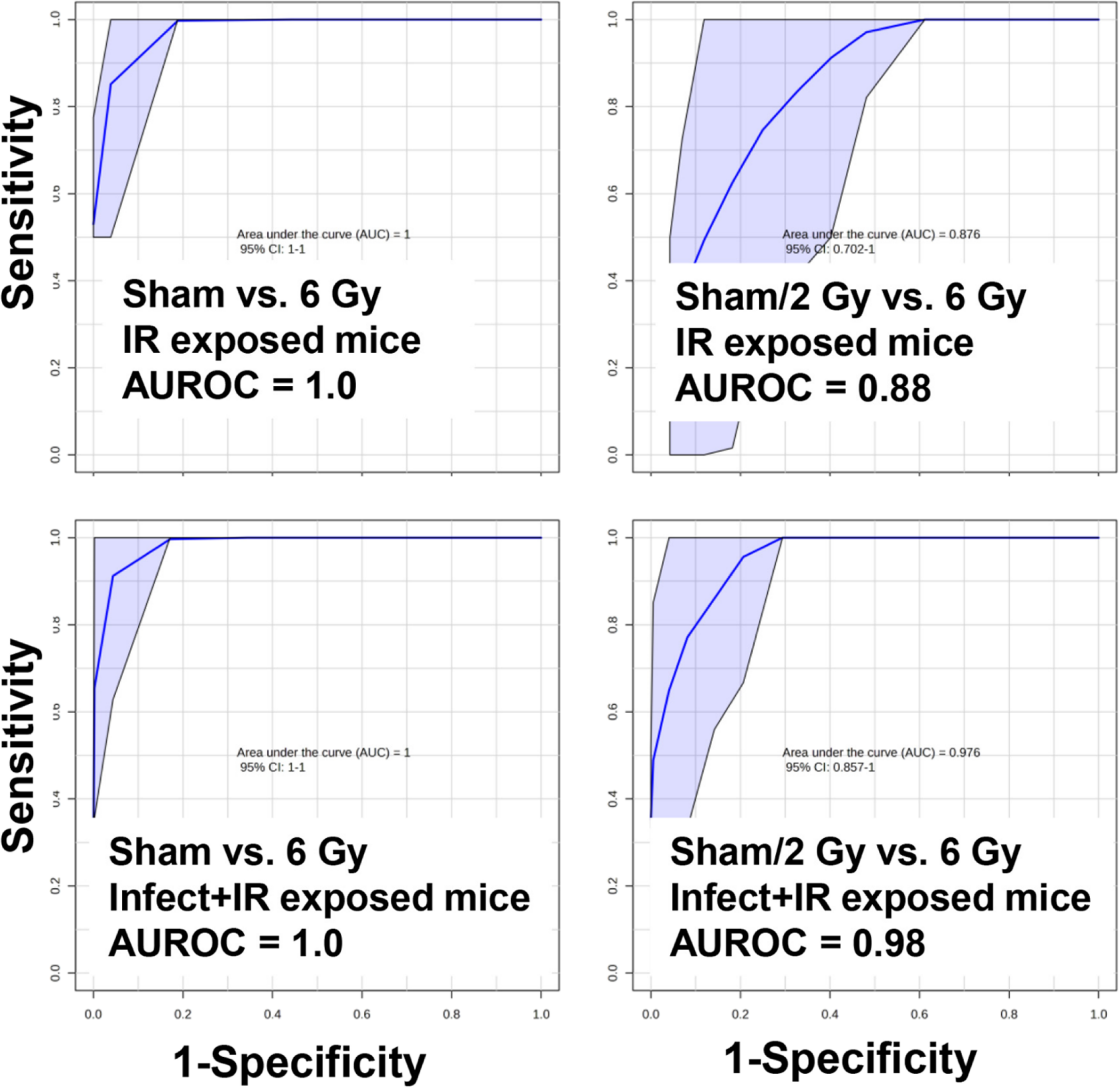
AUROC curves show that a combined urinary metabolite panel (carnitine, acetylcarnitine, propionylcarnitine, Hex-V-I, taurine, creatine, acetylspermidine, citric acid, azelaic acid, glutamic acid, and hexanoylglycine) can give excellent to very good sensitivity and specificity for individuals exposed to IR irrespective of the infection status (AUROC classification: excellent ≥ 0.9, very good ≥ 0.8).

### CBC and Radiation Metabolites in Serum

*Lm-OVA* infection led to increased WBC (*p* < 0.001), lymphocyte (LYM, *p* = 0.008), monocyte (MON, *p* = 0.008), and neutrophil (NEU, *p* < 0.001) counts (Figure 4). While IR exposure decreased WBC and LYM levels for both groups, we found elevated levels remaining in WBCs for infect + IR mice following 2 Gy (*p* = 0.010). No other significant differences were observed in CBC numbers between noninfect + IR and infect + IR mice following IR exposure. In resting (noninfected) conditions, the order of radiosensitivity is lymphocytes > neutrophils > monocytes. However, following *Lm-OVA* infection, neutrophils exhibited heightened sensitivity to radiation-induced cytotoxicity. Activated neutrophils generate high levels of reactive oxygen species (ROS) during the oxidative burst to kill bacteria, with the additional ROS generated by radiation further being exacerbated by oxidative stress. Moreover, the transcriptionally active state of these neutrophils renders their DNA more vulnerable to radiation-induced damage. Activated neutrophils are also primed for apoptosis due to activation-induced stress. Consequently, our observation of decreased neutrophil numbers is due to their increased susceptibility under the combined stress of the dual hit from infection and radiation.

**Figure 4:**
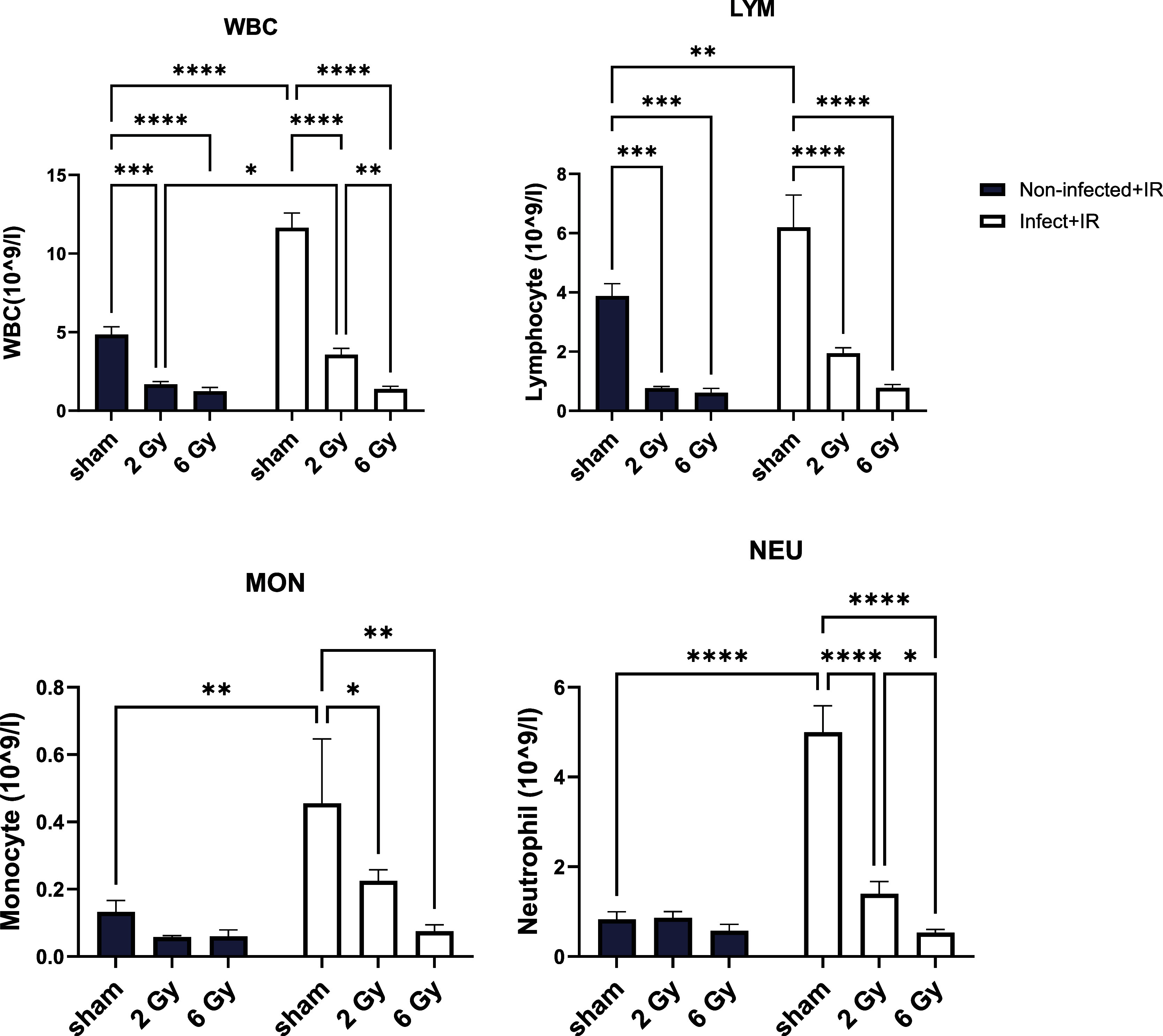
CBC analysis at 1 day following 2 or 6 Gy IR exposure alone or in combination with *L. monocytogenes* infection.

In serum, our initial data matrix was composed of 3863 spectral features in the ESI+ mode and 4648 spectral features in the ESI– mode. We observed more defined separation between serum groups compared to urine, primarily between the sham and 2 Gy TBI groups. Overall, unsupervised PCA analysis shows that irrespective of infection, the irradiated individuals can be clearly identified in a dose-dependent manner using either urine or serum, as has been previously shown across mammalian models (e.g., refs 48–50 ) (Figure 5).

**Figure 5:**
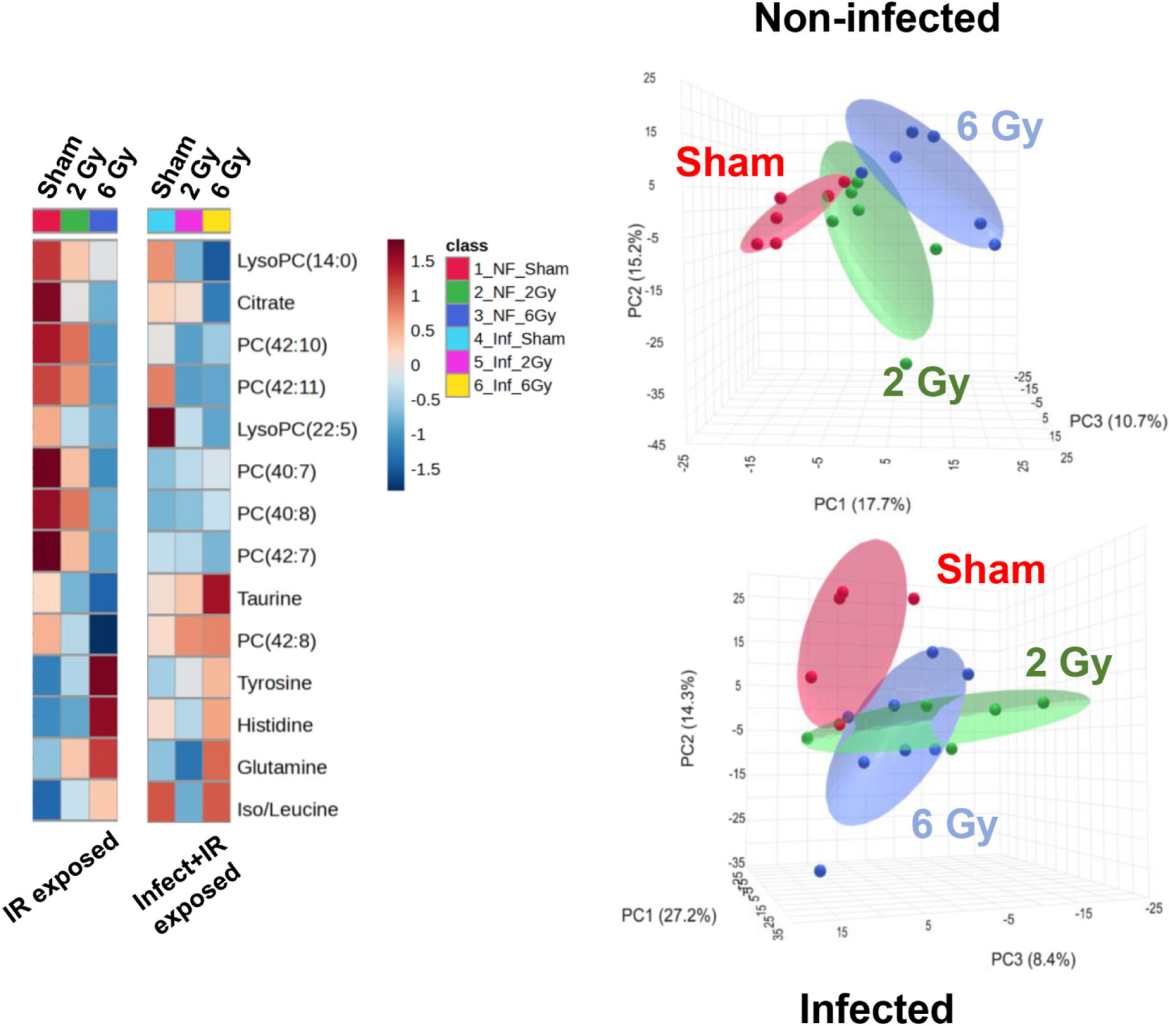
(Left) Heatmap showing fold changes of validated serum metabolites following IR exposure alone or in combination with *L. monocytogenes* infection. (Right) PCA of the combined ESI+ and ESI– data sets from untargeted metabolomic profiling. The sham, 2, and 6 Gy irradiated groups were all distinguished from each other irrespective of the infection status.

Blood is an important biofluid for measuring changes in lipid circulation, as the source and concentration of urinary lipids can be difficult to interpret.^51^ In noninfect + IR exposed mice, we found a reduction in serum PCs and LysoPCs, with up to a 50% reduced concentration following a 6 Gy exposure (Figures 5, S3, Table 3 and 4). Similar to the trends in urinary TCA cycle intermediates, reduced PC [40:7] *p* = 0.050, PC [40:8] *p* = 0.020, PC [42:7] *p* = 0.020, and PC [42:10] *p* = 0.020 levels were found in infect + sham mice compared to noninfect + sham mice, thus obscuring potential radiation effects. An exhaustive list of putative serum metabolites that were significantly changed due to infection alone is provided in Supporting Information File 1. As a facultative intracellular pathogen, *L. monocytogenes* pathogenesis can affect the host lipidome at several points, including cell entry through lipid rafts to the production of inflammatory mediators.^52^ Changes in the host lipidome have been documented for other types of bacterial infections. Decreased levels of PCs and LysoPCs in plasma^53^ and serum^54–56^ are characteristic of community-acquired pneumonia. Plasma LysoPC levels decrease during sepsis,^57^ although this is typically accompanied by decreased lipolytic activity (e.g., PLA2) and increased PC levels,^58,59^ as reflected in the PC/LysoPC ratio. Overall, general dyslipidemia is considered a hallmark of inflammatory effects downstream of cytokine production following several acute bacterial or parasitic infections.^60^ Interestingly, LysoPC (14:0), PC (42:8), and PC (42:11) levels remained unchanged due to *Lm-OVA* infection, making them robust IR markers. Both LysoPC (14:0) and (22:5) have been identified as IR metabolites in microbiome-depleted mice^29^ and a p38αß^Y323F^ mouse strain with attenuated inflammatory responses.^61^ Future research needs to be performed to determine if these lipids can be used as IR biomarkers following other bacterial infections.

**Table 3:** Validated Serum Metabolites

								MS/MS fragments		
metabolite	adduct	RT	experimental (*m*/*z*)	calculated (*m*/*z*)	mass error (ppm)	HMDB	formula	fragment 1	fragment 2	fragment 3
PC(40:7)	H+	8.69	832.5843	832.5856	1.6	^a^	C_48_H_82_NO_8_P	184.0730	104.1075	86.0959
PC(40:8)	H+	8.38	830.5685	830.5700	1.8	^a^	C_48_H_80_NO_8_P	184.0729	104.1099	86.0963
PC(42:7)	H+	9.08	860.6137	860.6169	3.7	^a^	C_50_H_86_NO_8_P	184.0720	104.1057	86.0961
PC(42:8)	H+	8.74	858.5994	858.6013	2.2	^a^	C_50_H_84_NO_8_P	184.0727	104.1049	86.0969
PC(42:10)	H+	8.28	854.5685	854.5700	1.8	^a^	C_50_H_80_NO_8_P	184.0733	104.1058	86.0935
PC(42:11)	H+	7.99	852.5529	852.5543	1.6	^a^	C_50_H_78_NO_8_P	184.0754	104.1068	86.0945
LysoPC(14:0)	H+	4.78	468.3076	468.3090	3.0	^a^	C_22_H_46_NO_7_P	184.0736	104.1071	86.0968
LysoPC(22:5)	H+	5.22	570.3552	570.3560	1.4	^a^	C_30_H_52_NO_7_P	184.0747	104.1053	86.0954
iso/leucine	H+	0.47	132.1019	132.1025	4.5		C_16_H_13_NO_2_	86.0967	69.0705	
glutamine	H–	0.28	145.0612	145.0613	0.7	0000641	C_5_H_10_N_2_O_3_	127.0505	109.0411	84.0455
histidine	H+	0.28	156.0777	156.0773	2.6	0000177	C_6_H_9_N_3_O_2_	110.072	95.0616	83.0636
tyrosine	H+	0.34	182.0815	182.0817	1.1	0000158	C_9_H_11_NO_3_	136.0756	119.0482	91.0538
citric acid	H–	0.34	191.0191	191.0192	0.5	0000094	C_6_H_8_O_7_	173.0063	111.0091	87.0087
taurine	H–	0.29	124.0076	124.0068	6.5	0000251	C_2_H_7_NO_3_S	106.9805	94.9788	79.9569

a Fragments from different lipid isomers are present and cannot be separated using the current LC method.

**Table 4:** Fold Changes and ANOVA *p* Values for Validated Serum Metabolites for IR-Exposed Noninfected or Infected Mice^a^

	irradiated						nonirradiated	
	*p* value		fold change				p value	fold change
	noninfected	infected	noninfected		infected		noninfected vs infected	
metabolite serum			2 Gy	6 Gy	2 Gy	6 Gy	sham	
PC(40:7)	<0.001	NS	0.8	0.6	1.1	1.1	0.050	0.6
PC(40:8)	0.001	NS	0.9	0.6	1.0	1.1	0.020	0.6
PC(42:7)	<0.001	NS	0.8	0.5	1.0	0.9	0.058	0.6
PC(42:8)	<0.001	NS	0.8	0.6	1.1	1.1	0.757	0.9
PC(42:10)	<0.001	NS	0.9	0.5	0.7	0.8	0.052	0.7
PC(42:11)	<0.001	0.001	0.8	0.3	0.3	0.3	0.556	0.9
LysoPC(14:0)	<0.001	<0.001	0.9	0.8	1.1	0.6	0.362	0.9
LysoPC(22:5)	<0.001	<0.001	0.7	0.5	1.2	0.3	0.038	1.4
iso/leucine	0.021	0.041	1.1	1.3	0.9	1.3	0.895	1.0
histidine	0.003	NS	1.0	1.3	0.9	1.0	0.109	1.1
glutamine	0.008	0.042	1.1	1.2	0.9	1.2	0.989	1.0
tyrosine	0.001	NS	1.1	1.5	1.0	1.2	0.556	1.1
citric acid	0.002	NS	0.7	0.5	0.9	0.6	0.374	0.7
taurine	0.033	NS	0.8	0.7	1.0	1.2	0.941	1.0

a Statistically significant differences between the sham and infect + sham groups were also compared.

Profiling circulating amino acid levels typically provides a richer panel compared to their excreted levels in urine. Changes in urinary amino acid levels were observed only for glutamic acid and taurine; however, both of these metabolites were useful for identifying irradiated individuals irrespective of the infection status. In serum, amino acid levels were not affected by *Lm-OVA* infection at 5 dpi in the current study (Tables 3 and 4), but it has been noted that differential effects occur during sepsis^62^ and pneumonia.^63^ Perturbed serum levels were observed in noninfect + IR animals for iso/leucine, glutamine, histidine, tyrosine, and taurine (Figures 5, S3, Tables 3 and 4). The effect in infect + IR-exposed animals was attenuated following *Lm-OVA* infection and was statistically significant only for iso/leucine (*p* = 0.041) and glutamine (*p* = 0.042). In addition to IR-attenuated responses due to bacterial infection, circulating amino acid levels may also be affected by radiation shielding. We previously found statistically significant changes in serum arginine, lysine, and tyrosine in mice that were exposed to high-dose-rate TBI and upper body irradiation, but no changes were observed in lower-body-irradiated mice.^20^ Although blood citrulline levels are well correlated with gastrointestinal injury,^64^ the sources of these amino acid changes, such as dietary influences, on predictive accuracy need further consideration.^65^

The top 10 serum metabolites for ROC curve construction included citric acid, LysoPC (14:0), LysoPC (22:5), PC (42:8), PC (42:11), leucine, glutamine, histidine, tyrosine, and taurine, which gave excellent sensitivity and specificity for all groups (Figure 6). AUROC values were at 100% for noninfect + sham vs noninfect + 6 Gy (AUROC = 1.0, CI = 1–1) and noninfect + sham/2 Gy vs noninfect + 6 Gy (AUROC = 1.0, CI = 1–1); however, serum profiles did show an attenuated response due to infection, as reflected in the ROC curves with infect + sham vs infect + 6 Gy (AUROC = 0.99, CI = 0.9–1) and infect + sham/2 Gy vs noninfect + 6 Gy (AUROC = 0.91, CI = 0.73–1).

**Figure 6:**
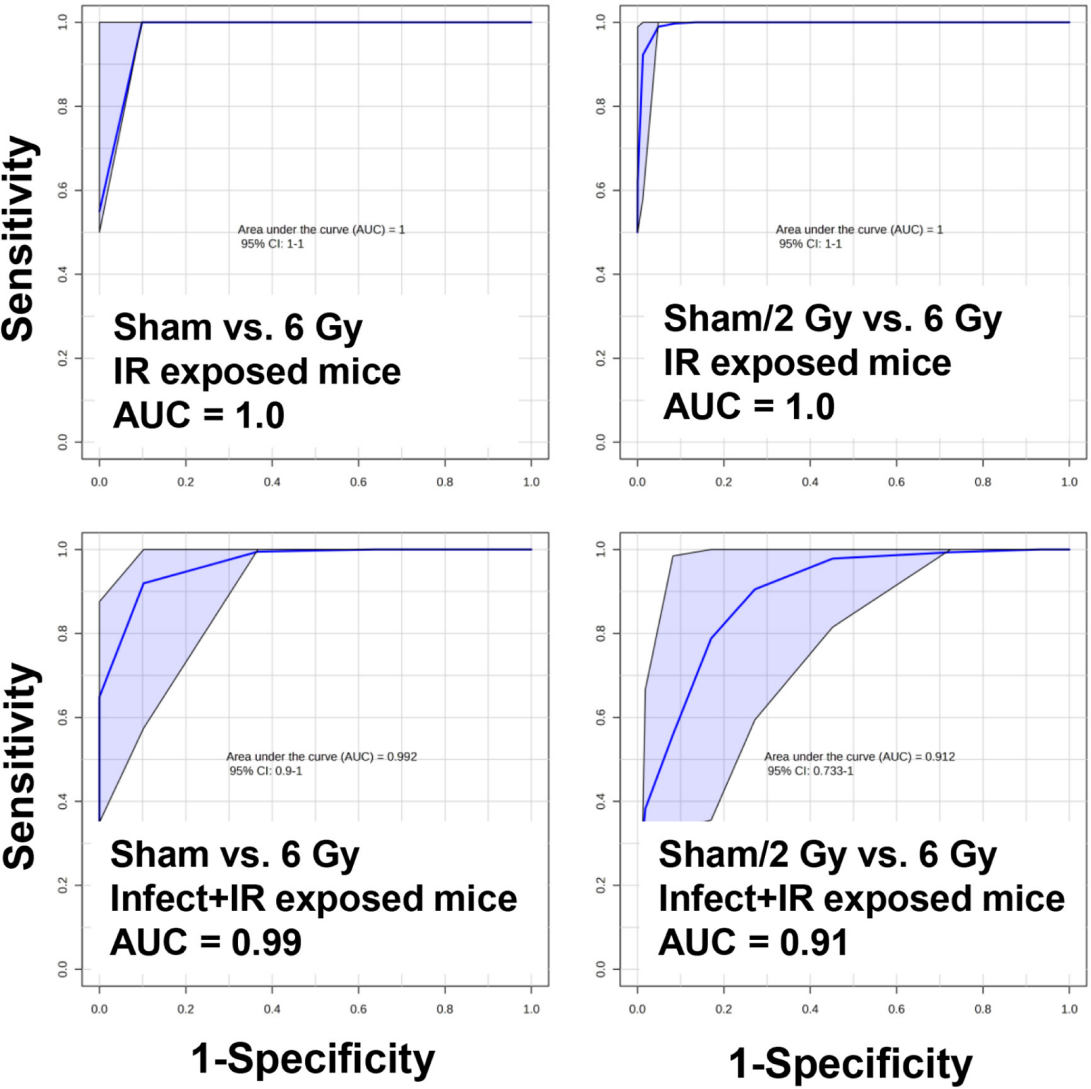
AUROC curves show that a combined serum metabolite panel (citric acid, LysoPC [14:0], LysoPC [22:5], PC [42:8], PC [42:11], leucine, glutamine, histidine, tyrosine, and taurine) can give excellent to very good sensitivity and specificity for individuals exposed to IR irrespective of the infection status (AUROC classification: excellent ≥ 0.9).

### Radiation Metabolites in Urine and Serum Combined

To identify key metabolic markers predictive of radiation dose and infection status in mice, we performed a multivariate random forest analysis using combined urine and serum metabolite profiles. The model was trained to jointly predict the radiation dose (as a continuous outcome) and infection status (as a binary classification), incorporating all validated metabolites in a single, unified framework. Feature importance was evaluated using permutation-based variable importance scores (in parentheses below with its respective metabolite), which quantify each metabolite’s relative contribution to predictive accuracy. The top 10 predictive metabolites included LysoPC (14:0) (0.254), LysoPC (22:5) (2.239), PC (42:8) (0.114), PC (42:11) (3.827), and citric acid (1.244) from serum as well as glutamic acid (0.119), creatine (0.252), propionylcarnitine (0.104), acetylspermidine (0.101), and hexanoylglycine (0.376) from urine. The model achieved a root-mean-squared error (RMSE) of 1.31 Gy for radiation dose prediction, indicating a typical prediction error just over 1 Gy and 90% classification accuracy for infection status (Figure 7). To the best of our knowledge, this is the first study to combine both urine and serum metabolites for radiation biodosimetry in a murine model using a multivariate random forest analysis. Although direct comparisons are limited, prior studies using single biofluids have reported RMSEs ranging from ∼1.5–2.5 Gy, suggesting improved predictive precision here.

**Figure 7:**
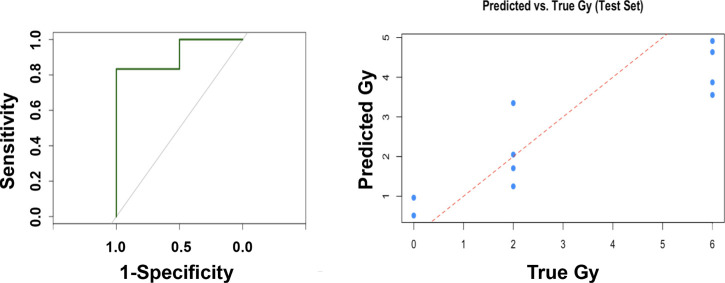
Multivariate random forest analysis was used to pick the top 10 radiation metabolites in urine and serum combined. The panel was composed of serum—LysoPC (14:0), LysoPC (22:5), PC (42:8), PC (42:11), citric acid and urine—glutamic acid, creatine, propionylcarnitine, acetylspermidine, hexanoylglycine (RMSE = 1.31 Gy, dose prediction = 90%).

### Study Limitations and Conclusions

With respect to study limitations, the primary pathogens of concern in the aftermath of a nuclear emergency will include endogenous intestinal microflora that can cause blood infections due to an impaired gut barrier and opportunistic exogenous pathogens that can cause pneumonia or colonize mucosal surfaces during a weakened immune state. *L. monocytogenes* is primarily a foodborne pathogen and will be of less concern in IR-exposed individuals; nevertheless, food contamination may still be an issue in a postexposure event. Additionally, real-world infections like sepsis and pneumonia are typically polymicrobial, while only one pathogen was used in the current study. Finally, the timing of opportunistic infections in a nuclear emergency scenario will likely follow the irradiation event. Future research will explore how additional murine models of sepsis will affect multiomic radiation biomarker panels and how complex exposures will play a role in this process.

The purpose of this study was to use the well-established *Lm-OVA* murine infection model to determine if bacterial infection combined with radiation injury could alter biofluid metabolite panels, which could affect dose reconstruction in the event of a radiological emergency. Not surprisingly, we found that combined IR exposures and infection can have profound effects on CBC levels and metabolite panels; however, small-molecule biomarkers can be useful to identify individuals needing medical care in an emergency situation. Future research is desperately needed to expand on these results using additional infectious agents, complex exposure scenarios, and medical treatments. Also, changes in other IR biomarkers, such as gene expression, during complex biological exposure systems need to be explored for constructing multiomic biodosimetry assays.

## Supplementary Material





## Data Availability

Metabolomics data have been uploaded to Metabolomics Workbench with Study ID ST004101 and ST004100 (https://www.metabolomicsworkbench.org/).

## Notes

Targeted pathway analysis of the validated urinary metabolites in the fatty acid oxidation, TCA cycle, and malate–aspartate shuttle pathways; normalized abundance values for urinary metabolites that were significantly different following IR exposure for either noninfected or *L. monocytogenes-*infected mice; and normalized abundance values for serum metabolites that were significantly different following IR exposure for either noninfected or *L. monocytogenes-*infected mice (PDF)
Putative serum metabolites that were significantly different between noninfected or *L. monocytogenes-*infected mice (XLSX)
