# Supplementary material for: Effects of Combined Bacterial Infection and Radiation Injury on Biofluid Metabolite Profiles in the Murine Model
Source: ACS Omega. 2025 Oct 15;10(42):49962–74. doi: 10.1021/acsomega.5c06273 (PMC12572980; doi:10.1021/acsomega.5c06273)
Supplement: Supplementary file 1 [file ao5c06273_si_001.pdf]

Effects of combined bacterial infection and radiation injury on biofluid metabolite profiles in the murine model

Evan L. Pannkuk[1,2,3*], Anika Kot[1], Lorreta Yun-Tien Lin[1], Igor Shuryak[4], Eric Wang[4], Albert J. Fornace Jr.[1,2,3], Heng-Hong Li[1,2]

[1]Department of Oncology, Lombardi Comprehensive Cancer Center, Georgetown University Medical Center, Washington, DC

[2]Department of Biochemistry and Molecular & Cellular Biology, Georgetown University Medical Center, Washington, DC

[3]Center for Metabolomic Studies, Georgetown University, Washington, DC

[4]Center for Radiological Research, Columbia University Irving Medical Center, New York, NY

Corresponding Author

*Evan L. Pannkuk, PhD

Georgetown University, 3970 Reservoir Road, NW, New Research Building, Room E504, Washington, DC, USA, 20007

E-mail: elp44@georgetown.edu, Phone: (202) 687-5650

Supplemental Figure 1. A targeted pathway analysis of the validated urinary metabolites fell in the fatty acid oxidation, TCA cycle, and malate-aspartate shuttle pathways.

Supplemental Figure 2. Normalized abundance values for urinary metabolites that were significantly different following IR exposure for either non-infected or *Listeria monocytogenes* infected mice.

Supplemental Figure 3. Normalized abundance values for serum metabolites that were significantly different following IR exposure for either non-infected or *Listeria monocytogenes* infected mice.

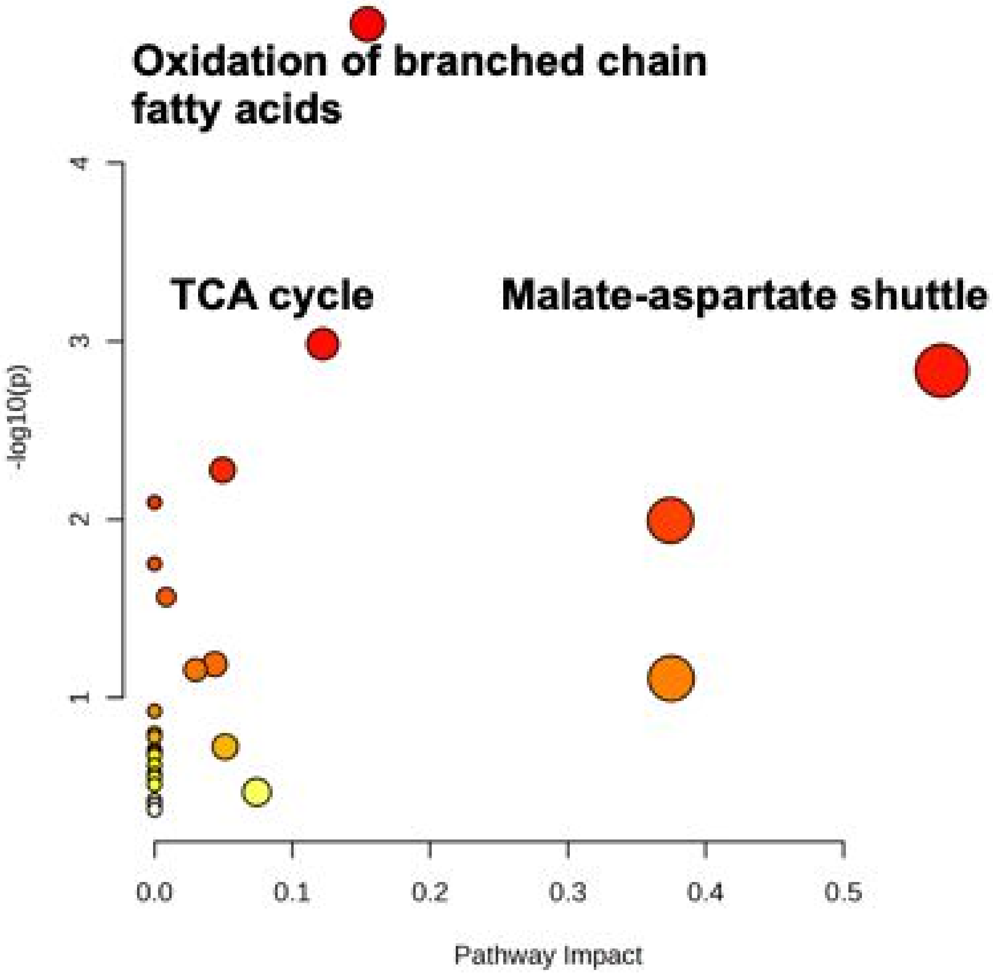

Supplemental Figure 1. A targeted pathway analysis of the validated urinary metabolites fell in the fatty acid oxidation, TCA cycle, and malate-aspartate shuttle pathways.

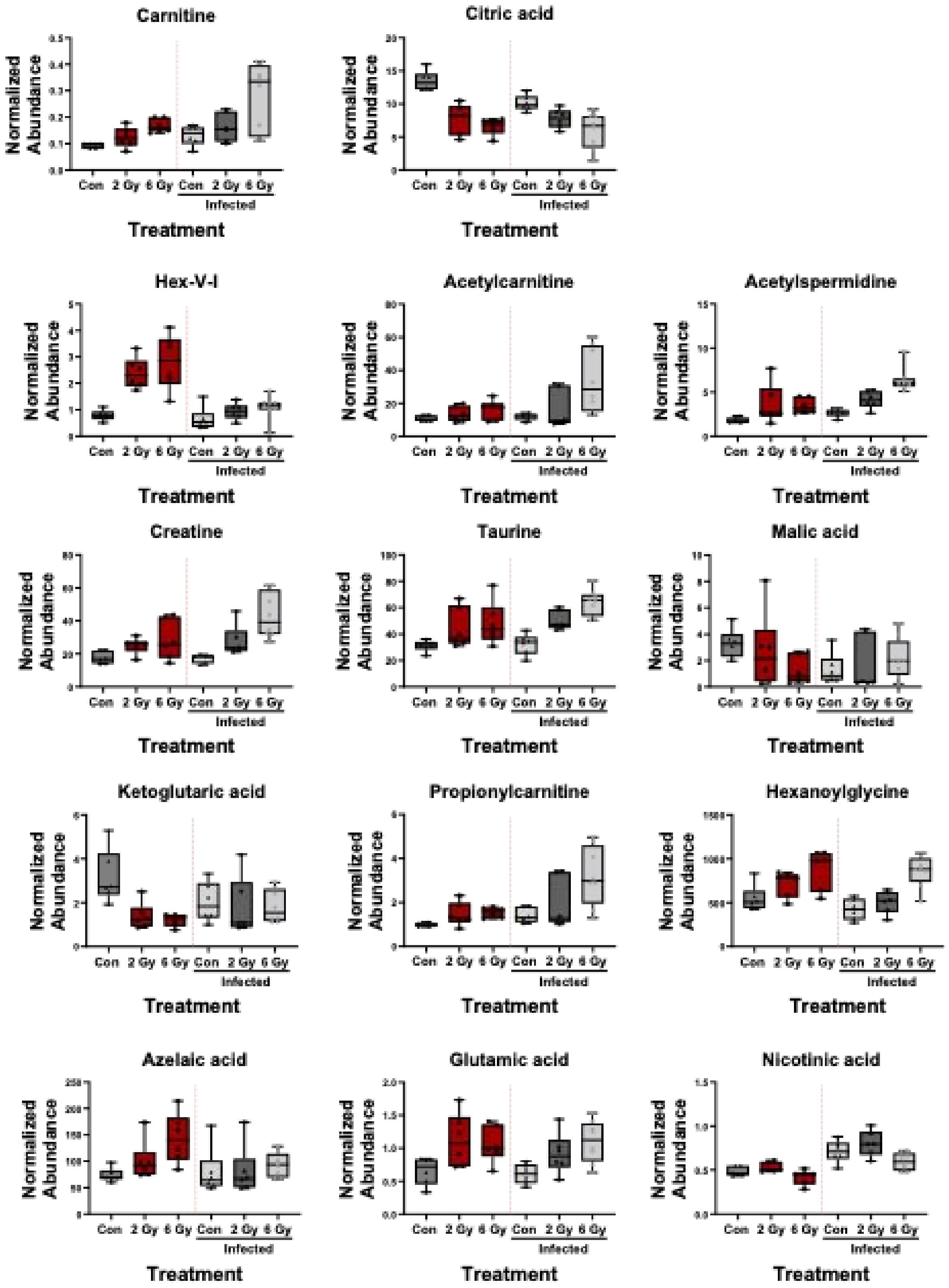

Supplemental Figure 2. Normalized abundance values for urinary metabolites that were significantly different following IR exposure for either non-infected or *Listeria monocytogenes* infected mice.

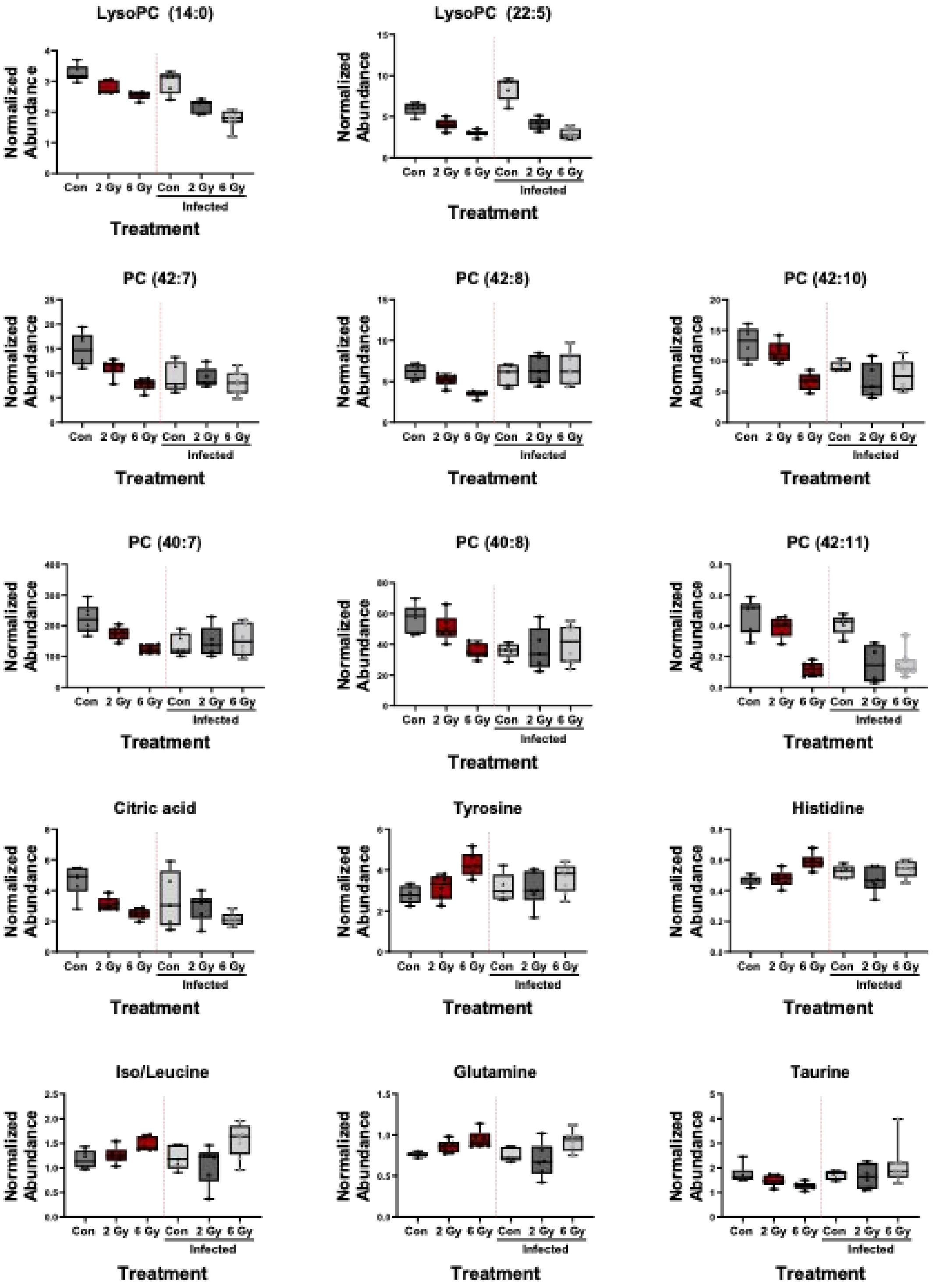

Supplemental Figure 3. Normalized abundance values for serum metabolites that were significantly different following IR exposure for either non-infected or *Listeria monocytogenes* infected mice.
